# Microbial and Physicochemical Dynamics of Kocho, Fermented Food from Enset

**DOI:** 10.1155/2023/6645989

**Published:** 2023-10-19

**Authors:** Dereba Workineh Seboka, Abay Tabor Bejiga, Debela Jufar Turunesh, Andualem Arimo Turito, Abayeneh Girma

**Affiliations:** ^1^Department of Biology, College of Natural and Computational Science, Mizan-Tepi University, P.O. Box. 121, Tepi, Ethiopia; ^2^Department of Chemistry, College of Natural and Computational Science, Mizan-Tepi University, P.O. Box. 121, Tepi, Ethiopia; ^3^Department of Biology, College of Natural and Computational Science, Mekdela Amba University, P.O. Box. 32, Tuluawlia, Ethiopia

## Abstract

Over 20 million Ethiopians depend on enset (*Ensete ventricosum*) as a staple or costaple food. “Kocho,” “Bulla,” and “Amicho” are the three main food types obtained from enset. This review aimed to summarize the physicochemical and microbial dynamics of kocho. It is the most common food obtained from the scraped pseudostem and decorticated corm of enset after a long period of fermentation. The quality of kocho depends on the maturity of the enset plant, the enset processing method, the fermentation period, and the dynamics of microorganisms during the fermentation process. Microorganisms play a significant role in kocho fermentation to enhance its nutritional quality, improve sensory properties, and reduce spoilage and disease-causing agents. The populations of microbes available in kocho fermentation include lactic acid bacteria (LAB), Enterobacteriaceae, acetic acid bacteria (AAB), yeasts and molds, and *Clostridium* spp., which have both positive and negative impacts on kocho quality. There is a visible variation in microbial dynamics during kocho fermentation caused by the fermentation period. As the fermentation day increases, species of LAB also increase, whereas counts of Enterobacteriaceae decrease. This is due to a decrease in pH, which leads to an increase in titratable acidity. Moisture content also slightly decreases as fermentation progresses. Dynamics in the microbial population and physicochemical parameters ensure the development of desirable qualities in kocho and enhance the acceptability of the final product. Organic acids (such as lactic acid, acetic acid, and propionic acid), bacteriocins, phenolic compounds, flavonoids, and tannins are bioactive compounds produced by microorganisms during Kocho fermentation. Further research is needed on the molecular identification of microorganisms during Kocho fermentation.

## 1. Introduction

Enset (*Ensete ventricosum*), also referred to as a false banana, is a food security crop that provides staple or costaple food for more than 20 million people in Ethiopia [1, 2]. Enset is a monocarpic, perennial, and monocotyledonous plant that originated and was domesticated in Ethiopia [3]. Geographically distributed as a wild plant species in many parts of Sub-Saharan Africa and Asia, it is commonly cultivated as a food crop only in the Ethiopian highlands [3, 4]. Enset plants are grown in a relatively wide range of environmental conditions and soil types [3, 5]. The plant possesses deep root systems that enable it to withstand a longer period of drought (about 5 months) than other crops [2]. The plant is accessible throughout the year and has the capability to serve a larger number of people in the future as staple and costaple foods. Recently, the government of Ethiopia started a new project on enset cultivation and adaptation to the Amhara and Tigray regions [6].

*Kocho*, *Bulla*, and *Amicho* are the main food products obtained from the enset plant after processing. Kocho is a fermented food obtained from the scraped pseudostem and pulverized corm of the plant [7]. *Kocho* is consumed as a staple or costaple food in central, southern, and southwestern parts of Ethiopia [8]. It is rich in carbohydrates, minerals, phenolics, and fibers [9]. However, all food products prepared from enset are poor in protein, fat, and vitamin content [9, 10]. Enset-based food should be consumed with meat, cheese, peas, or beans to supplement proteins [2, 9, 11].

The variety of enset plants, processing approaches, and fermentation periods influence the quality of kocho [8, 12]. Microbes play a significant role in the fermentation process of traditional foods, which can contribute to improved nutritional quality and organoleptic characteristics, enhance shelf-life, and be used to increase and standardize the fermentation process [13]. The dynamics of microbes are used in the fermentation process for the metabolism of starch, protein, and lipid, which results in an overall change in its nutritional and sensory quality [14]. The proliferation of some microbes under favourable conditions compromises kocho qualities such as taste, color, texture, or aroma [7, 9, 15]. The overall change in nutritional and sensory quality of kocho has resulted from microbial profiles inside the fermenting enset mass [9, 14, 15]. LAB, Enterobacteriaceae, acetic acid bacteria, yeasts, and molds, as well as *Clostridium* spp., are some of the microbial load groups presented in fermenting kocho [7, 8].

Studies by Hunduma [16] and Elifu [17] showed LAB as a dominant microbial group in the entire enset fermentation process. This is due to their tolerance to an acidic environment [18, 19], and hence lactic acid-producing bacteria are suggested to be the potential starter culture organisms for enset fermentation [17, 19, 20]. However, reviews of related literature on physicochemical parameters and microbial dynamics of fermenting enset mass are not analyzed in depth or well organised at all. This review summarizes the microbial and physicochemical dynamics of fermenting enset mass and their roles during fermentation.

## 2. Enset Plant (*Ensete ventricosum*)

### 2.1. Brief Description of Enset Plant

**Figure 1:**
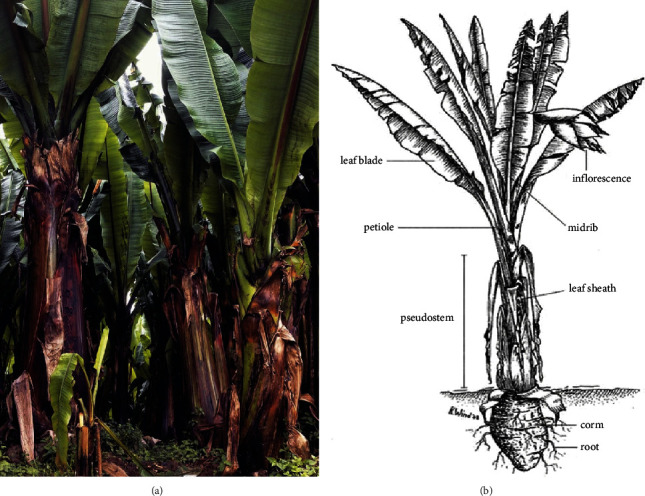
Enset plant (a) and its parts (b).

Ethiopia is a homeland for many cultivated plant crops such as teff (*Eragrostis teff*), coffee (*Coffee arabica*), and enset (*Ensete ventricosum*) [21]. Different scholars have developed theories that argue for Ethiopia's origin and domestication of the enset plant before 10,000 years ago [4]. Enset, also known as *Ensete ventricosum*, is a common plant that belongs to the root and tuber group. Enset is a member of the genus *Enset*, the family *Musaceae*, and the order *Scitaminae* [22–24]. Taxonomically, enset plants are categorized under the order *Zingiberales* of the *Musaceae* family and the genus *Ensete* [25, 26]. Morphologically, the plant reaches 4−8 m [26, 27]. It is a tall perennial herbaceous root crop that grows to a height of 4 to 11 metres [13]. It has long (5 m) and broad (0.75 to 1.5 m) spiral leaves and a bulky pseudostem that is 2 to 5 metres long. Adventitious is its root system. A corm, which is 0.70–1.8 metres in length and 1.5–2.5 metres in circumference when fully grown, makes up the plant's underground section [23, 24]. Flowers are unisexual; female flowers grow closer to the centre of the inflorescence, while male flowers grow farther away. The fruits are fibrous, oblong-obovate, 8–15 cm × 3–4.5 cm, and become orange when fully grown (Figure 1). Enset is distributed as a wild plant species in many parts of Sub-Saharan Africa, central, and eastern Africa and Asia [4, 5, 28] and is only cultivated as a food crop in Ethiopia, mainly in the southern and southwestern parts of the country [4]. Seasonal droughts in the central and northern parts of Ethiopia lead to the expansion of enset cultivations to other parts of Ethiopia [25, 29].

### 2.2. Utilization of Enset Plant in Ethiopia

In the southern, southwestern, and central parts of the Ethiopian highlands, enset is used as food for humans, fodder for animals, for other uses such as industrial fibre, rob material in fences, and house building, for mattresses and seat making, local packaging material, and a substitute for table plates [4, 6, 30, 31]. It stabilizes soils and microclimates and has valuable cultural importance [3]. Fibre is produced while the enset leaf sheath is decorticated [31, 32]. The fibre in foods also has a prominent role in lowering blood glucose and blood fat levels [9]. The vigorous pseudostem, corm, and inflorescence stalk are used to produce nutritious food for humans and fodder for animals [33]. Foods obtained from enset have divergences in preparation techniques and consumption patterns [10].

*Amicho*, *Bulla*, and *Kocho* are the main foods prepared from the enset plant [2]. Amicho is the fleshy inner portion of the enset corm, which is cooked and eaten separately and has a similar taste to potato [27]. The corm is also used as a source of kocho, and it acts as a fermenting agent or starter called g*amma* or g*amancho* (undefined and locally prepared starter culture from enset corm) [9, 12]. Bulla is an enset-based food obtained through the squeezing process of pulverized kocho and consumed as a staple food [30, 34]. Bulla food is considered the best quality enset-based food and is mainly produced from a fully matured enset plant [27]. Enset-based food products are rich in carbohydrates and minerals such as calcium and potassium, which is very crucial for consumers, but it lacks many other nutrients such as vitamins and only contain a fewer amount of fat and proteins [10].

### 2.3. Traditional Kocho Fermentation

Traditional kocho fermentation requires a variety of equipment and ingredients for processing as well as for accelerating its duration time. It is varied from place to place both in equipment used for processing and in ingredients required for facilitating the fermentation process [15]. The first step in the process of kocho fermentation is the collection of matured enset plants carried out by experienced women [13, 35]. After a mature enset plant has been selected, leaves are removed, cleared, and dried leaf sheaths from the plant, and the surrounding parts are prepared for the next steps [15]. The dug-up underground corm is detached and cleaned to isolate the true stem from the root. Then, the inner leaf sheaths are separated from the pseudostem down to the real stem, which is a segment between the corm and the pseudostem [36]. The true stem is isolated from the underground corm [12]. The pseudostem and corm of the enset plant are scraped and pulverized, respectively [36].

**Figure 2:**
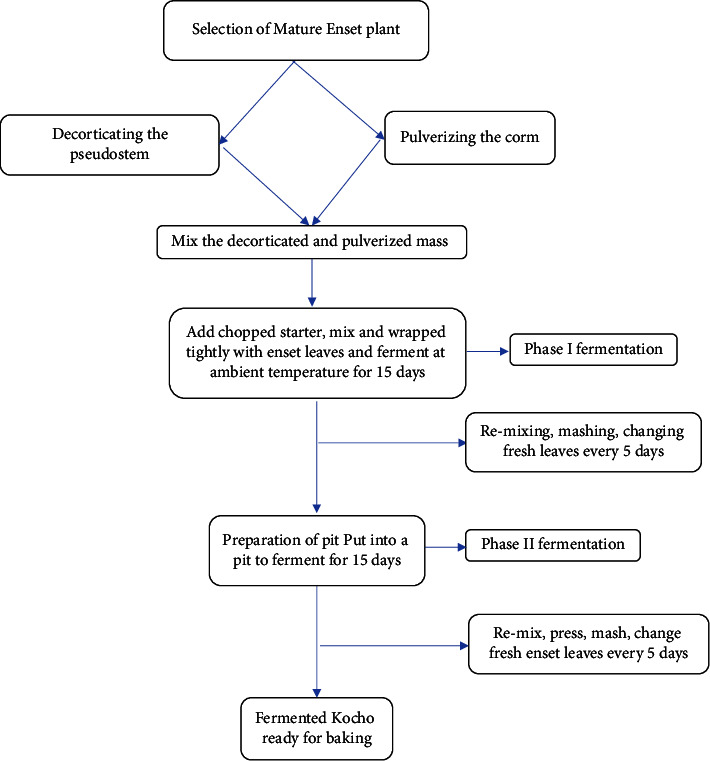
A flowchart of traditional kocho fermentation process.

Fermentation starts after kocho is stored in an earthen pit, which may range from three months to one year for completion [20, 35, 37]. It depends on the climatic conditions of that environment [15]. In warmer regions, fermentation is rapid and may terminate within 15 days to at least one month [38], while in the cooler regions, it is kept in a pit for years [15]. The traditional processing of enset has two phases (Figure 2): phase one (surface fermentation), the beginning of fermentation which is continued for about 15 days, and phase two (pit fermentation) [35]. Kocho fermentation methods and times are different from location to location [36].

### 2.4. Fermented Enset

Kocho is a traditionally fermented and indigenous starch-rich food product in Ethiopia that is prepared from enset plants [7, 36]. Next to injera and wheat bread, it is a popular fermented food consumed in Ethiopia [39]. Scraped pseudostem, pulverized corm, and decorticated enset pulp are mixed and fermented to produce carbohydrate-rich kocho [13, 35, 37]. It is widely consumed by millions of Ethiopians [2] and plays a crucial role in ensuring food security [25]. The bread prepared from fermented enset is known as kocho bread [37]. Bosha et al. [9] reported that kocho is rich in carbohydrates. However, it has low protein content [10, 12]. Kocho production requires a long period of fermentation which is prolonged from three to six months [37, 40].

The quality of kocho food depends on the age of the harvested enset plant, accession type [25], harvesting season, and fermentation period [12]. Moreover, within one plant, the quality is influenced by the part of the leaf sheath and corm processed [27].

### 2.5. Physicochemical Dynamics during Enset Fermentation

The physicochemical properties investigated during kocho fermentation have great potential to enhance the quality of the final product. Moisture content, titratable acidity, pH, and fermenting temperatures (Table 1) are considered physicochemical parameters, and their dynamics during kocho fermentation play an indispensable role in determining the final quality of kocho.

#### 2.5.1. Moisture Content

Water is essential for the growth and metabolism of fermenting microbes found in kocho dough [35]. Kocho fermented with boiled decorticated enset pulp has a higher moisture content but is low in protein, carbohydrate, fibre, and fat content as compared with kocho fermented with nonboiled decorticated enset pulp [12]. Mohammed et al. [11] showed that enset contains a huge amount of water, which ranges between 85 and 90%. As kocho fermentation time is extended, the moisture content decreases, probably due to excessive leaching during pit fermentation and in different steps in the preparation of kocho [9, 35]. The moisture content of kocho generally declined as the time of fermentation increased [8, 13]. These are the characteristics of fermenting microbes that lead to the reduction of moisture within the fermentation time [35]. Enset variety and fermentation periods play a prominent role in determining the amount of moisture in the kocho [12].

#### 2.5.2. Temperature

Hunduma and Mogessie [15] reported that the internal temperature of unfermented kocho remains below 20°C while that of fermented kocho ranges from 19 to 23°C. This report was in line with the findings by Karssa et al. [35], who showed an increment in the internal temperature from 19 to 24°C. Finally, in the pit fermentation, the internal temperature of the kocho varied between 20.5 and 26.1°C. Andeta et al. [8] reported the internal temperature of fermenting enset biomass as 22.5 ± 0.2°C for the pit, 22.7 ± 0.2°C for the erosa (a bamboo basket made from enset leaf sheath), and 19.3 ± 0.1°C for jar fermentation.

#### 2.5.3. pH

Kocho fermentation within the pit resulted in a decrease in pH and an increase in titratable acidity [8, 35]. Similarly, Andeta et al. [13] reported that the pH value of kocho is influenced by the fermentation methods. A similar finding also reported by Yirmaga [12] showed that as the titratable acidity of kocho increased, the pH value decreased. LAB and yeasts are dominant species during the fermentation of kocho since they are acid-tolerant and can survive at low pH [15]. Karssa et al. [35] reported that the pH of fermented enset inoculated with a traditional starter culture (*gamancho)* is lower than that of treatments fermented without starter culture during fermentation periods. Their difference indicated that treatment with traditional starter cultures accelerates the fermentation time by providing high numbers of fermenting microbes.

#### 2.5.4. Titratable Acidity

According to Andeta et al. [8], titratable acidity was predominantly increased through fermentation periods for pits and jars in different amounts. The titratable acidity of jar fermentation was higher than that of pit and erosa fermentations, which is in line with pH evolution. At the start of enset fermentation, the amount of titratable acidity was lower as compared with the later time of fermentation due to the increased population of acid-producing microorganisms [37]. Yirmaga [12] reported that the amount of titratable acidity increased significantly from 10 to 13 days of the fermentation period. Concomitantly, as compared to the 10^th^ and 30^th^ days of fermentation, the amount of titratable acidity is high on the 30^th^ day of fermentation. This reflects that enset fermentation is an acidic-rich environment that allows the reduction of pH and increment of titratable acidity. Karssa et al. [35] reported that the increment in titratable acidity of fermenting kocho dough is favouring the growth and activities of lactic acid bacteria.

### 2.6. Microbial Dynamics during Enset Fermentation

Microbial dynamics are the driving force for the traditional fermentation process, which provides the desired quality of fermented food [7]. They play a major role in traditional food fermentation to enhance nutritional quality, extend shelf-life, and contribute to the palatability and wholesomeness of the product [13, 41]. Enterobacteriaceae, lactic acid bacteria, yeasts, and molds are the predominant microbial groups reported during kocho fermentation (Table 2), and some of them are used as starters or initiators of the fermentation process [7, 8].

#### 2.6.1. Acetic Acid Bacteria

Acetic acid bacteria are a group of Gram-negative bacteria with great potential for oxidizing ethanol to acetic acid [46]. Through a long period of fermentation, AAB isolated from the LAB selective media, which may increase the number of metabolites and sensory attributes, maintain hygiene, and promote wholesome kocho fermentation [7, 42]. Concomitantly, higher counts of AAB were isolated from fermenting kocho after inoculating the LAB starter culture at the onset of fermentation [19, 20]. Aeration causes the enhancement of AAB in fermenting kocho mass, which may affect the sensory quality of fermented mass [7, 9, 42]. Organic acids produced during fermentation are used as a preservative for fermented food since they have the potential to produce acetic acid, lactic acid, and bacterocins [8, 30, 47].

#### 2.6.2. Enterobacteriaceae

The Enterobacteriaceae are microorganisms with great potential for disease-causing activity, encompassing beneficial commensal microbiota, opportunistic pathogens that can inflict considerable morbidity and mortality on compromised hosts, and principal pathogens capable of initiating illness in individuals in perfect health [48]. Enterobacteriaceae are the indicator bacteria for the microbiological quality of food and the hygiene status of a production process and pose a microbiological risk for consumers [49]. Counts of Enterobacteriaceae are relatively higher at the beginning of the fermentation process and reach an undetectable level at the final stages of Phase II fermentation. This is due to the reduction of pH and an increase in titratable acidity [8, 35]. The growth of Enterobacteriaceae is also inhibited by increasing fermentation periods of kocho, except for *Escherichia coli* (*E*. *coli*) O157:H7, which can grow in an acidic environment [50]. This reflects the fact that *E. coli* O157:H7 can survive in low PH (around 5.00) medium [50, 51]. The counts of Enterobacteriaceae remained below the detection limit on day 7 and other subsequent sampling days for both uninoculated and inoculated Kocho samples with LAB starter inoculant [52]. This might be due to the acidic environment created by endogenously present or added LAB, which creates unfavorable conditions for the Enterobacteriaceae [9, 13].

The decline in the counts of Enterobacteriaceae plays a profound role in enhancing kocho safety and eliminating pathogenic microbes such as mold [37, 53]. Counts of Enterobacteriaceae were reduced to below the detectable level after day 1 for the pits and jars fermentation and after day 7 for the basket fermentation [8]. Likewise, Karssa et al. [35] and Andeta et al. [13] reported counts of Enterobacteriaceae below the detection limit between 12 and 15 days during the fermentation of kocho due to adverse conditions and a reduction of pH over fermentation periods. This might have resulted from unfavorable conditions occurring during the fermentation process and the production of bacterocins and other antibacterial substances by some lactic acid bacteria [7, 8]. In the traditional enset fermentation system, counts of Enterobacteriaceae were detected until 30 days of fermentation [35, 37].

#### 2.6.3. Lactic Acid Bacteria (LAB)

LAB is a group of heterogeneous bacteria that play a great role in different fermentation processes (Table 2). They can ferment carbohydrate-containing foods and produce lactic acid as a byproduct of fermentation [54]. It constitutes a maximum proportion of total microbial counts and dominates an entire enset fermentation system [7, 8, 35]. To enhance the fermentation process, inoculation of starter culture which is used though the starter should exclude and compete with the natural microbe [55]. The use of starter culture reduces the duration of fermentation and allows its completion in a short period of time [19, 36].

LAB contributes to the stability and safety of kocho by inhibiting pathogenic and spoilage bacteria [17]. They provide metabolites such as lactic acid, acetic acid, carbon dioxide, ethanol, hydrogen peroxide, and antimicrobial peptides [56]. Metabolite compounds produced by LAB have greatly improved the flavour and odour of kocho products [7]. LABs carry out a biochemical conversion of carbohydrates into organic or lactic acid and other metabolites during fermentation [57]. It contributes to the stability and safety of kocho by inhibiting pathogenic and deteriorative bacteria [17]. They are used as starter culture, a product with high viable microbial counts, and when added to certain foods, they accelerate fermentation, leading to a final product with the desired change in aroma, texture, and flavour profile [19, 41]. To enhance the fermentation process, inoculation of starter culture is used, though the starter should exclude and compete with the natural microbe [55]. The use of starter culture reduces the duration of fermentation and allows its completion in a short period of time [19, 36].

Andeta et al. [19] and Weldemichael et al. [36] revealed promising strains of LAB suitable for starter culture for enset fermentation. Strains of *Lactobacillus plantarum* and *Leuconostoc mesenteroides* are validated as potential starter inoculum for enset fermentation [19]. *Leuconostoc* and *Lactococcus* spp. are the most abundant LAB in the initial phases of the fermentation, while *Lactobacillus, Weissella*, and *Bifidobacterium* spp. were dominant at the later stages of fermentation [8]. *L. mesenteroides* occurred dominantly at the onset of kocho fermentation and is used as an initiator for the fermentation process, while *Prevotella paludivivens*, *Lactobacillus sp*., and *Bifidobacterium* are less abundant species at the beginning of fermentation [13].

As the number of LAB increases during kocho fermentation, the sugar content of kocho decreases due to an increase in the fermentation period [9, 58]. In kocho fermentation, species of LAB, mainly *L. mesenteroides*, are responsible for decreasing moisture content and pH while increasing titratable acidity [13]. This is might be the heterofermentative characteristics of *L. mesenteroides* that allow the production of carbon dioxide, ethanol, and organic acids [59], as cited in [60]. This reflects the fact that they are acid-producing microorganisms [19]. LAB present in kocho fermentation is also useful for the development of volatile compounds, which enhance the sensory quality of kocho products [36]. The findings of Karssa and Papini [63] also revealed similar results: an increment in LAB counts allows for a reduction in pH value and an increase in titratable acidity. Strains of LAB starter culture are responsible for the development of kocho quality [20]. The mutual interaction of *L. plantarum* and *L. mesenteroides* during kocho fermentation causes the degradation of starch content, reduction of pH value, and increase in titratable acidity that inhibit pathogenic microbes and improve the desirable organoleptic characteristics of kocho [42].

All microbial counts, including LAB, decreased as fermentation progressed [8, 35]. Dibaba et al. [37] also revealed similar findings that reflect the increase in LAB count until 30 days of fermentation and its decline at 60 days of fermentation. This might be the low pH value's inhibitory effect on the microbes inside the fermenting system [13, 38].

#### 2.6.4. Yeasts and Molds

Yeast and molds are a large and diverse group of food-borne microbes that include several hundred species that cause various degrees of deterioration and decomposition on foods [62]. Identification and characterization of yeast species in kocho fermentation are useful in improving nutritional quality, maintaining hygiene, and ensuring the safety of kocho products [7]. Yeast has potential to break down sugars into acid, carbon dioxide gas, and other flavour compounds during vegetable fermentation. Moreover, the acid produced during fermentation provides vegetable tartness and inhibits pathogenic microbes' growth [63]. Identified species of yeast such as *Filobasidilla neoformans*, *Candida*, and *Trichosporon* are harmful microbes to the health of human beings [42]. Therefore, safety and hygienic requirements are important during enset processing periods [14]. The characterization of yeast inside kocho fermentation is crucial for the formulation of starter culture and for improving, standardizing, and modernizing the quality of traditional enset fermentation and preparation [14].

Enough amount of oxygen is required for the growth of yeast during the fermentation of kocho [7]. However, as counts of yeast increase during Kocho fermentation, starch degradation also enhances and imparts antimicrobial metabolites such as ethanol, organic acids, and vitamins, which play a crucial role in the growth of LAB [43]. Counts of yeast and mold slightly decrease as fermentation days proceed to end in kocho fermentation [52]. This could result from the anaerobic conditions of the fermenting enset mass. This is in agreement with the finding of Dibaba et al. [37] that revealed counts of yeast and mold were reduced due to anaerobic conditions of *gamma* fermentation or a lack of sufficient oxygen during fermentation periods.

#### 2.6.5. *Clostridium* Spores

*Clostridium* is composed of a large spectrum of Gram-positive, mesophilic, and anaerobic species. The *Clostridium* sp. bacteria acts in various environments, providing agro, ecological benefits in plant growth promotion and participation in industrial processes, and replacing in both cases chemicals harmful to the environment [64]. Counts of anaerobic *Clostridium* spores increased as fermentation days increased for different enset accessions [13]. Inoculation of LAB starter inoculum inhibits *Clostridium* spores' growth during enset fermentation more than control or uninoculated cultures [19]. *Clostridium* species play a great role in incrementing pH values during enset fermentation. Proteolytic *Clostridia* were known to breakdown amino acids into ammonia and thereby increase the pH [8, 44, 45]. Supportably, it was shown that saccharolytic *Clostridia*, *Clostridium tyrobutyricum,* increased the pH of a fermenting enset [60]. *Clostridium* spores increased during kocho fermentation [7].

### 2.7. Bioactive Compounds Produced by Microorganisms during Kocho Fermentation

During kocho fermentation, organic acids such as lactic acid, acetic acid, and propionic acid are among the bioactive substances that are produced [7]. During the fermentation process, acetic acid bacteria (AAB) and lactic acid bacteria (LAB) create these organic acids. Bacteriocins are antimicrobial peptides that LAB also makes; they prevent the growth of harmful bacteria [36]. Other bioactive substances such as phenolic compounds, flavonoids, and tannins are also produced during kocho fermentation in addition to organic acids and bacteriocins [42]. The antioxidant qualities of these substances can aid in preventing diseases brought on by oxidative stress.

## 3. Conclusion

Enset is a plant resource with significant potential to provide a starch-rich food called *Kocho*. It also contains low protein and mineral content in terms of nutritional quality and is a staple or costaple food in terms of food security. The dynamics of the microbial population and changes in physicochemical parameters during enset fermentation ensure the quality of the product. There is a microbial population in *Kocho* fermentation that provides desired sensory character, improves nutritional quality, enhances product shelf-life, and eliminates pathogenic agents. Monitoring microbial and physicochemical dynamics inside fermenting kocho results in good-quality products of enset-based food.

## Figures and Tables

**Table 1:** Physicochemical dynamics and their roles in fermenting enset mass.

S. no.	Product	Physicochemical parameter	Their status and roles during fermentation	References
1	Fermenting kocho/enset mass	pH	(i) The pH of fermenting enset mass/kocho decreases as fermentation days increase (ii) It creates an acidic environment that creates unfavorable conditions for pathogenic microorganisms and inhibits them	[8, 15, 35]
		Titratable acidity	(i) Titratable acidity increases as fermentation days increase, resulting from a decrease in pH (ii) An increment in titratable acidity favours the growth of lactic acid bacteria	[12, 35]
		Moisture content	(i) The moisture content of fermenting enset/kocho declined as the fermentation day progressed (ii) A decrease in moisture content allows an increment in the dry matter of kocho	[8, 9, 11, 13]
		Temperature	(i) The internal temperature increases as fermentation day increases, but it depends on the external temperature	[8, 35]

**Table 2:** Microbial dynamics and their roles in fermenting enset mass.

S. no.	Product	Microbial dynamics	Their status and roles during fermentation	References
1	Fermenting kocho/enset mass	Acetic acid bacteria	(i) Increase as fermentation days increase (ii) Aeration favours the growth of AAB (iii) Flavors produced by AAB are used as a preservative for fermented mass	[7, 9, 20, 42]
		Lactic acid bacteria	(i) Increase at the onset and middle of the fermentation day and decrease as the fermentation day proceeds to end (ii) Produce acidic metabolites that allow the reduction of pH (iii)Used as a starter culture for enset fermentation	[19, 20, 35]
		Enterobacteriaceae	(i) Counts of Enterobacteriaceae decreased as fermentation days increased (ii) The acidic environment created in fermentation mass allows the reduction of Enterobacteriaceae counts	[8, 35]
		Yeast and molds	(i) Yeast and mold count slightly decrease as fermentation proceeds to an end (ii) They play a crucial role in degrading starch and imparting antimicrobial metabolites that allow the growth of lactic acid bacteria	[8, 43]
		*Clostridium* spores	(i) Anaerobic *Clostridium* increased as fermentation days increased (ii) They enhance the breakdown of amino acids into ammonia, which results in a reduction in pH	[13, 19, 44, 45]

## Data Availability

All data generated and analyzed during this study are included in this article.
